# Toward Tailored EUS‐Guided Gallbladder Drainage in the Era of Lumen‐Apposing Metal Stent

**DOI:** 10.1111/den.70216

**Published:** 2026-06-30

**Authors:** Shuntaro Mukai, Takao Itoi

**Affiliations:** ^1^ Department of Gastroenterology and Hepatology Tokyo Medical University Tokyo Japan

Laparoscopic cholecystectomy remains the standard treatment for acute cholecystitis. However, gallbladder drainage is recommended for patients considered to be at high surgical risk because of poor general condition, advanced age, comorbidities, or underlying malignancy. Percutaneous transhepatic gallbladder drainage (PTGBD) has long been regarded as the standard drainage technique because of its high technical and clinical success rates. In recent years, however, endoscopic internal drainage, including endoscopic transpapillary gallbladder drainage (ETGBD) and endoscopic ultrasound‐guided gallbladder drainage (EUS‐GBD), has become increasingly widespread [1].

The first case of EUS‐GBD using double‐pigtail plastic stent (DPPS) was reported in 2007, and EUS‐GBD using fully covered metal stents (FCMS) began to appear after 2011 [2, 3]. Although these early approaches established the feasibility of transmural gallbladder drainage, stent placement was technically demanding and was associated with concerns regarding migration, maldeployment, and bile leakage. The introduction of lumen‐apposing metal stent (LAMS) represented a major advance. Since the first report of EUS‐GBD using LAMS in 2012, technical success and safety have improved substantially [4]. In particular, electrocautery‐enhanced one‐step LAMS delivery systems have simplified the procedure and have become a preferred option for EUS‐GBD in many expert centers [5].

Recent multicenter data from the United States using electrocautery‐enhanced LAMS showed excellent outcomes, with a technical success rate of 99.1%, clinical success rate of 97.2%, and procedure‐related adverse event rate of only 3.7% [6]. These results clearly demonstrate the clinical value of LAMS for EUS‐GBD. In addition, an international multicenter randomized controlled trial comparing EUS‐GBD with PTGBD in very high‐risk surgical patients with acute cholecystitis reported that EUS‐GBD was associated with lower rates of adverse events, 30‐day reintervention, and recurrent cholecystitis, as well as less postprocedural pain [7]. A meta‐analysis comparing EUS‐GBD with ETGBD also showed higher technical and clinical success rates with EUS‐GBD [8]. Although the overall adverse event rate did not differ significantly between the two approaches, recurrent cholecystitis was less frequent after EUS‐GBD. Furthermore, a network meta‐analysis by Podboy et al. highlighted the limitations of each drainage strategy: PTGBD is associated with a higher need for reintervention and unplanned hospitalization, whereas ETGBD remains technically challenging, with success rates of approximately 80%–90% even when various technical modifications are used [9]. These findings suggest that EUS‐GBD is particularly suitable for patients who are unlikely to undergo cholecystectomy after gallbladder drainage because of poor general condition, comorbid disease, or advanced malignancy.

LAMS has several theoretical advantages over conventional DPPS and FCMS. They are short, large in diameter, and equipped with bilateral flanges that provide strong lumen apposition and reduce the risk of migration. These features make LAMS highly attractive for EUS‐GBD. However, currently available LAMS also have important anatomical limitations. The commonly used saddle length is approximately 10 mm; therefore, cases with a long distance between the gastrointestinal wall and gallbladder lumen, or marked gallbladder wall thickening, may be at risk of stent maldeployment or subsequent buried stent syndrome. Moreover, because of the characteristics of the delivery system and distal flange deployment, safe placement generally requires an adequately distended gallbladder lumen. In patients with an anatomically unfavorable gallbladder, such as a collapsed gallbladder after PTGBD, severe wall thickening, or a long puncture distance, FCMS placement may still be selected for EUS‐GBD.

In this issue of *Digestive Endoscopy*, Chuncharunee et al. describe a novel, simplified EUS‐GBD technique using a 19‐gauge Franseen‐tip fine‐needle biopsy needle and a modified slim FCMS [10]. The key concept of this approach is to combine the cutting ability of the Franseen‐tip needle with a 5.9‐Fr slim delivery system. Because the three‐plane cutting tip of the Franseen needle can create a relatively large cylindrical puncture tract, the puncture itself may function as tract dilation. As a result, the stent can be advanced and deployed without additional fistula dilation. This is an important technical advantage because tract dilation is one of the steps that can increase the risk of bile leakage, guidewire dislodgement, and procedural complexity. Another unique feature of this technique is the modification of the distal portion of the FCMS. Before deployment, the authors created multiple side holes in the covering membrane of the distal 15‐mm segment of the stent, which is intended to be positioned within the gallbladder lumen. This modification is designed to enhance intragallbladder drainage and maintain drainage even if partial stent kinking occurs after gallbladder decompression. In their single‐center retrospective study, the authors applied this technique to 18 consecutive patients with acute cholecystitis associated with malignant biliary obstruction who were considered unsuitable for LAMS placement. Technical and clinical success were achieved in all patients. During a median follow‐up period of 6 months, only one patient developed recurrent cholecystitis, which was successfully managed endoscopically. No severe adverse events or 30‐day mortality occurred. This study is clinically interesting because it suggests that EUS‐GBD can be tailored according to anatomical conditions, rather than being performed with a single standard device in all patients. LAMS should remain the first‐line device when the gallbladder is sufficiently distended, the puncture distance is short, and stable apposition between the gallbladder and gastrointestinal wall can be obtained. However, the technique proposed by Chuncharunee et al. may be a useful alternative in patients with LAMS‐unfavorable anatomy. It may also be applicable when internalization is desired after PTGBD in patients with a collapsed gallbladder. In addition, because this technique does not require electrocautery, it may have potential advantages in selected patients with a bleeding tendency, although this hypothesis requires further evaluation.

Nevertheless, several limitations should be acknowledged. This was a single‐center retrospective study including only 18 patients, and the median follow‐up period was 6 months. Therefore, the reproducibility, learning curve, long‐term stent patency, risk of late recurrent cholecystitis, and safety of long‐term indwelling modified FCMS remain unclear. Furthermore, this technique was evaluated mainly in patients with malignant biliary obstruction at a high‐volume center with substantial experience in EUS‐GBD. Whether similar outcomes can be achieved in other settings or by less experienced endoscopists requires prospective multicenter validation.

The evolution of EUS‐GBD has entered the LAMS era, but this does not mean that LAMS is optimal for every patient. Rather, the next step may be to establish a tailored EUS‐GBD strategy in which LAMS, FCMS, DPPS, and transpapillary approaches are selected according to patient background, gallbladder anatomy, expected prognosis, and the need for long‐term management. The technique reported by Chuncharunee et al. provides an important reminder that innovation in EUS‐GBD should not only pursue procedural simplicity, but also expand the range of patients who can safely benefit from internal gallbladder drainage. Further studies with larger cohorts and longer follow‐up are warranted to clarify the optimal positioning of this modified FCMS technique in relation to EUS‐GBD using LAMS.

## Author Contributions

Shuntaro Mukai: drafting of the article. Takao Itoi: critical revision of the article for important intellectual content.

## Funding

The authors have nothing to report.

## Conflicts of Interest

Author S.M. has received honoraria for lectures from Boston Scientific Japan. Author T.I. is a consultant for Boston Scientific Japan.

## Linked Articles

This article is linked to Chuncharunee et al. papers. To view this article, visit https://doi.org/10.1111/den.70152.

## Data Availability

Data sharing not applicable to this article as no datasets were generated or analysed during the current study.
